# Emergent Endovascular Management of Carotid Blowout Syndrome: A Case Report

**DOI:** 10.7759/cureus.81098

**Published:** 2025-03-24

**Authors:** Elias Salloum, Ahmed Altan

**Affiliations:** 1 Interventional Radiology, Moffitt Cancer Center, Tampa, USA

**Keywords:** carotid blowout, catheter angiogram, embolization, external beam radiation, pseudoaneurysm

## Abstract

Carotid blowout syndrome is a rare but life-threatening complication in heavily treated head and neck cancer patients whose outcome depends on early diagnosis and prompt intervention. This report describes the case of a patient with prior irradiation who underwent emergent endovascular treatment after rupture of a 2.9cm external carotid artery branch pseudoaneurysm.

## Introduction

Carotid blowout syndrome (CBS) is a rare but serious complication in heavily treated head and neck cancer patients and carries a high rate of mortality without appropriate diagnosis and intervention [1-6]. Arterial injury from prior surgery or radiation may result in arterial wall necrosis and pseudoaneurysm formation, which can result in catastrophic hemorrhage [7]. This report illustrates the case of a patient who underwent prior irradiation requiring emergent angiography and intervention after acute rupture of a 2.9cm external maxillary artery pseudoaneurysm.

## Case presentation

**Figure 1:**
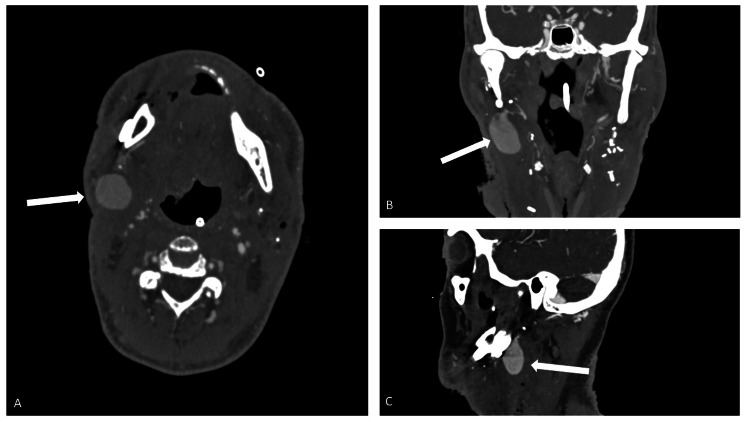
Axial (A), coronal (B), and sagittal (C) images of a neck CT angiogram demonstrating a 2.9cm pseudoaneurysm from the external maxillary artery. The pseudoaneurysm sac extends inferolaterally into the right neck post-surgical bed.

The patient is a 63-year-old man with a history of squamous cell carcinoma of the right tonsil for which he received 70 Gy radiation therapy with concurrent cisplatin chemotherapy nearly 12 years prior to presentation. More recently, he developed osteoradionecrosis of the right mandible and was found to have a pathologic fracture along with an orocutaneous fistula. He subsequently underwent right neck dissection, right segmental mandibulectomy with the placement of hardware, tracheotomy, and fibula free flap and skin graft three weeks prior to presentation. He was seen at an outside hospital after experiencing sudden “sentinel” bleeding from the right neck, which was treated with pressure and packing having only required one unit of blood. He was transferred to our facility for further evaluation. Upon arrival, he was hemodynamically stable. A CT angiogram of the neck was ordered, which demonstrated a 2.9cm external carotid artery branch pseudoaneurysm extending into the surgical bed (Figure 1).

Shortly after imaging was performed, the patient coughed and began to hemorrhage from his right neck and oral cavity. Despite manual compression, the patient rapidly became hypotensive ultimately requiring vasopressor support and endotracheal intubation for airway protection. He was emergently taken to Interventional Radiology for endovascular evaluation and management.

Upon arrival to the angiography suite, the patient’s systolic blood pressure was noted to be 40mmHg via arterial line. The Otolaryngology service was present and assisted in compression and management of the actively bleeding neck wound.

**Figure 2:**
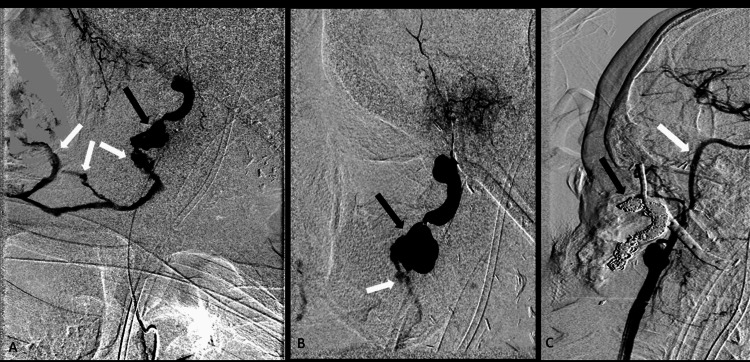
(A, B) DSA in sagittal oblique projection demonstrating pseudoaneurysm sac (black arrow) from the right external maxillary artery. There is active extravasation (white arrow) from the sac and extending into the right neck soft tissues and oral cavity. (C) Post-coil embolization DSA demonstrating a coil pack within the pseudoaneurysm sac and extending into the right external maxillary artery (black arrow). Note that the internal carotid artery (white arrow) and external carotid artery remain widely patent. DSA, digital subtraction angiogram

From a right common femoral artery approach, angiography of the right common carotid artery demonstrated diminutive arterial caliber likely related to prior irradiation. A pseudoaneurysm was identified originating from the distal right internal maxillary artery, one of the terminal branches of the external common carotid artery, with active extravasation into the surgical bed and oral cavity (Figure 2).

A microcatheter was used to select the right internal maxillary artery and angiography was performed, further localizing the pseudoaneurysm. Coil embolization was performed in the pseudoaneurysm sac utilizing a combination of detachable and pushable microcoils until complete occlusion. Careful efforts were made to completely embolize the internal maxillary artery while sparing the parent external carotid artery. Post-intervention angiography from the common carotid artery demonstrated occlusion of the pseudoaneurysm and preserved patency of the internal carotid artery and external carotid artery and its proximal branches.

After the embolization, the patient did not experience recurrent hemorrhage and no longer required vasopressor support. He remained intubated for surgery the following day where the Otolaryngology team performed surgical evacuation of the right neck hematoma, excisional debridement of right intraoral flap tissues, and flap and skin graft reconstruction.

The remainder of the hospital course was uneventful, and the patient was discharged a few days later. The patient underwent appropriate follow-up, as determined by the surgical team, and has no active disease without further intervention.

## Discussion

CBS is an uncommon but devastating complication in patients being treated for head and neck cancers. CBS results in the setting of arterial wall necrosis, which can occur following radiation, surgical resection, tumor invasion, or a combination of these factors. The overall incidence of CBS after major oncologic surgery of the head and neck ranges from 3% to 4.5% [1-6]. The incidence in patients who have received prior irradiation is as high as 21.1% [7]. Overall, prior radiotherapy has been administered in 80%-90% of patients who experience CBS [1,2,4,5,8]. Chen et al. reported that patients who receive a total radiation dose of >70 Gy to the neck incurred an almost 14-fold increased risk of developing CBS [9]. Rupture of the carotid artery arises in the common carotid artery near the bifurcation in 60-70% of cases and usually occurs 10-40 days after surgery [8]. Delayed hemorrhage can also occur more than two to three months after surgery [10]. Radiation therapy produces free radicals, which may cause vascular injury, weakening of the arterial wall, and subsequent pseudoaneurysm formation. Pseudoaneurysms have been reported 2-20 years after radical neck dissection and irradiation [11].

Complete angiography of the supra-aortic arteries is predominantly performed via transfemoral arterial approach to identify and characterize the vascular injury. Typical angiographic findings include arterial wall irregularity, pseudoaneurysm, and arterial wall rupture with active extravasation.

The degree and type of vascular injury can be graded on angiographic findings. Chang et al. classified injury according to the following findings: grade 1, no angiographic vascular disruption; grade 2, focal irregularity of the diseased artery; grade 3, pseudoaneurysm of the injured artery; and grade 4, active extravasation from ruptured artery [12].

Endovascular management has emerged as the preferred method to treat CBS. Surgical management can be difficult due to previously irradiated and/or potentially infected tissues. For these reasons, ligation of the common carotid artery/internal carotid artery is necessary in 7%-32% of patients with CBS treated surgically [8-9]. Emergency open surgery may also result in poor outcomes including local wound infection, flap necrosis, and global cerebral ischemia. An endovascular approach offers the advantage of localizing the exact site of injury, allowing precise treatment planning and intervention. For example, injury to the common carotid artery can often be treated with placement of a covered stent, thereby reducing the risk of occlusion and ensuing cerebral ischemia. Surgical vessel ligation is associated with a higher risk of cerebral ischemic complications. In a study of 45 patients, Lu et al. reported mortality rates of 10% and 28.6% for endovascular therapy and surgical ligation, respectively [6]. In our patient, the origin of the pseudoaneurysm was the maxillary branch of the external carotid artery, facilitating safe selective coil embolization and significantly reducing the risk of cerebral ischemia.

## Conclusions

CBS is a life-threatening emergency requiring urgent diagnosis and treatment. Endovascular management has emerged as an effective first-line therapy, allowing tailored treatment and reducing the need for open surgery and proximal arterial ligation.
